# Identification of antibody targets associated with lower HIV viral load and viremic control

**DOI:** 10.1371/journal.pone.0305976

**Published:** 2024-09-17

**Authors:** Wendy Grant-McAuley, William R. Morgenlander, Ingo Ruczinski, Kai Kammers, Oliver Laeyendecker, Sarah E. Hudelson, Manjusha Thakar, Estelle Piwowar-Manning, William Clarke, Autumn Breaud, Helen Ayles, Peter Bock, Ayana Moore, Barry Kosloff, Kwame Shanaube, Sue-Ann Meehan, Anneen van Deventer, Sarah Fidler, Richard Hayes, H. Benjamin Larman, Susan H. Eshleman, for the HPTN 071 (PopART) Study Team

**Affiliations:** 1 Department of Pathology, Johns Hopkins University School of Medicine, Baltimore, Maryland, United States of America; 2 Institute for Cell Engineering, Johns Hopkins University School of Medicine, Baltimore, Maryland, United States of America; 3 Department of Biostatistics, Johns Hopkins Bloomberg School of Public Health, Baltimore, Maryland, United States of America; 4 Quantitative Sciences Division, Department of Oncology, Sidney Kimmel Comprehensive Cancer Center, Johns Hopkins University School of Medicine, Baltimore, Maryland, United States of America; 5 Department of Medicine, Johns Hopkins University School of Medicine, Baltimore, Maryland, United States of America; 6 Laboratory of Immunoregulation, National Institute of Allergy and Infectious Diseases, National Institutes of Health, Baltimore, Maryland, United States of America; 7 Zambart, University of Zambia School of Public Health, Lusaka, Zambia; 8 Clinical Research Department, London School of Hygiene and Tropical Medicine, London, United Kingdom; 9 Desmond Tutu TB Center, Department of Paediatrics and Child Health, Stellenbosch University, Stellenbosch, Western Cape, South Africa; 10 FHI 360, Durham, North Carolina, United States of America; 11 Department of Infectious Disease, Imperial College London, London, United Kingdom; 12 Department of Infectious Disease Epidemiology, London School of Hygiene and Tropical Medicine, London, United Kingdom; "INSERM", FRANCE

## Abstract

**Background:**

High HIV viral loads (VL) are associated with increased morbidity, mortality, and on-going transmission. HIV controllers maintain low VLs in the absence of antiretroviral therapy (ART). We previously used a massively multiplexed antibody profiling assay (VirScan) to compare antibody profiles in HIV controllers and persons living with HIV (PWH) who were virally suppressed on ART. In this report, we used VirScan to evaluate whether antibody reactivity to specific HIV targets and broad reactivity across the HIV genome was associated with VL and controller status 1–2 years after infection.

**Methods:**

Samples were obtained from participants who acquired HIV infection in a community-randomized trial in Africa that evaluated an integrated strategy for HIV prevention (HPTN 071 PopART). Controller status was determined using VL and antiretroviral (ARV) drug data obtained at the seroconversion visit and 1 year later. Viremic controllers had VLs <2,000 copies/mL at both visits; non-controllers had VLs >2,000 copies/mL at both visits. Both groups had no ARV drugs detected at either visit. VirScan testing was performed at the second HIV-positive visit (1–2 years after HIV infection).

**Results:**

The study cohort included 13 viremic controllers and 64 non-controllers. We identified ten clusters of homologous peptides that had high levels of antibody reactivity (three in gag, three in env, two in integrase, one in protease, and one in vpu). Reactivity to 43 peptides (eight unique epitopes) in six of these clusters was associated with lower VL; reactivity to six of the eight epitopes was associated with HIV controller status. Higher aggregate antibody reactivity across the eight epitopes (more epitopes targeted, higher mean reactivity across all epitopes) and across the HIV genome was also associated with lower VL and controller status.

**Conclusions:**

We identified HIV antibody targets associated with lower VL and HIV controller status 1–2 years after infection. Robust aggregate responses to these targets and broad antibody reactivity across the HIV genome were also associated with lower VL and controller status. These findings provide novel insights into the relationship between humoral immunity and viral containment that could help inform the design of antibody-based approaches for reducing HIV VL.

## Introduction

HIV viral load usually peaks near the time of seroconversion and decreases as HIV-specific immune responses develop [1, 2]. Most people living with HIV (PWH) establish a viral load set point shortly after infection that reflects the balance between ongoing viral replication and immune clearance [1–6]. This set point is usually stable during chronic HIV infection but can vary widely between persons [1, 2, 7]. Higher viral loads are associated with increased HIV-related morbidity and mortality [8–15] and increased on-going transmission [12, 16, 17]. Effective antiretroviral treatment (ART) reduces HIV viral load to low levels, improving clinical outcomes [18–23] and reducing transmission risk [24–28].

Some PWH can control viral replication in the absence of ART. These individuals are often classified as elite or viremic controllers based on viral load measurements obtained at least one year apart (elite controllers: <50 copies/mL, viremic controllers <2,000 copies/mL) [29–32]. Use of a cutoff of 2,000 copies/mL for viremic controllers was suggested by Pereye and colleagues in 2007 [29], based on studies that demonstrated that viral loads <1–2,000 copies/mL were associated with slower disease progression and reduced HIV transmission [33–35]. HIV control develops in the early stages of infection [30, 36, 37]; some controllers have fewer symptoms in early infection compared to non-controllers [36, 38, 39]. HIV control is also associated with slower disease progression and reduced HIV-related mortality [30, 40]. The mechanisms responsible for HIV control are poorly understood and appear to involve complex interactions between viral and host factors [29, 32, 40]. Improved understanding of these mechanisms could inform development of immune-based interventions for HIV prevention and treatment.

Most research on HIV control has focused on the role of cellular immunity [29, 32, 40]. A role for humoral immunity in HIV control was generally dismissed following early studies that found lower titers of HIV-specific antibodies and neutralizing antibodies among controllers [29, 41–46], consistent with findings in non-controllers that antibody responses are less robust when PWH are virally suppressed on ART [47–50]. However, recent studies have identified controllers who have higher levels of antibody dependent cellular cytotoxicity (ADCC) [51], broadly neutralizing antibody (bNab) responses [52–54], and isotype diversity with associated polyfunctionality [55–59]; more robust responses against broad targets in HIV gag have also been observed in controllers [55, 60–62].

VirScan is a massively multiplexed assay that can be used to quantify antibody responses to peptide targets across the HIV genome [47, 63]. In prior study that included longitudinal samples collected from 14 days to 8.7 years after HIV infection, we found that antibody breadth (i.e., the number of unique epitopes targeted) increased early in infection and then declined or stabilized. Persons who had a decline in antibody breadth 9 months to 2 years after were more likely to start antiretroviral treatment (ART). In addition, a faster decline in antibody breadth was associated with a shorter time to ART initiation [44].

In a subsequent study, we used VirScan to characterize the fine specificity of HIV antibody responses in persons with established HIV infection [64]. That study identified seven clusters of homologous peptides that represented the primary antibody targets in both viremic controllers and non-controllers who were not on ART [64]. The study also found that higher levels of antibody reactivity to a target in gag p17 were associated with reduced plasma viral load [64]. The participants evaluated in that study had HIV infection of unknown duration. Because antibody titer, avidity, and breadth vary in persons infected for different periods of time [2, 47], differences in infection duration among the study participants may have confounded the results of that study.

In this report, we extended our prior work by characterizing antibody responses in a cohort of PWH who were known to be infected for 1–2 years and explored whether specific patterns of antibody reactivity were associated with low viral load and HIV control. This cohort included viremic controllers and non-controllers who acquired HIV infection during the HIV Prevention Trials Network (HPTN) 071 (PopART) trial [65]. This report used an unbiased approach to identify peptides that are more frequently targeted in HIV controllers and persons with lower viral loads. Findings from this study could support future research evaluating whether specific HIV antibodies play a causative role in viral containment.

## Methods

### Source of samples

Samples and data were obtained from the HPTN 071 trial (NCT 019000977), which demonstrated that universal delivery of a comprehensive HIV prevention package was associated with lower HIV incidence [66]. Plasma samples were collected annually from >48,000 adult participants from 21 communities in Zambia and South Africa [66] where most HIV infections are caused by subtype C HIV [67]. This report evaluated a subset of the 978 participants who acquired HIV infection during the trial (seroconverters) [65] and had controller status determined based on viral load and antiretroviral (ARV) drug testing [68]. Participants included in this report had samples and data available from at least three consecutive annual visits (one negative, two positive). Participants classified as controllers had viral loads <2,000 copies/mL with no ARV drugs detected at both HIV-positive visits; this method for identifying controllers is consistent with methods used in prior studies [29, 31, 69]. Participants classified as non-controllers had viral loads ≥2,000 copies/mL with no ARV drugs detected at both HIV-positive visits. VirScan testing was performed using samples collected at the second HIV-positive study visit (1–2 years after HIV acquisition). The analysis of HIV-1 VARscores included additional participants who were virally suppressed on antiretroviral therapy (ART; viral loads <400 copies/mL with ARV drugs detected at both HIV-positive visits).

### Laboratory methods

Laboratory testing was performed at the HPTN Laboratory Center (Johns Hopkins University, Baltimore, MD). HIV viral load was measured with the RealTime HIV-1 Viral Load assay (Abbott Molecular, Des Plaines, IL) using a validated dilution method (limit of quantification [LOQ]: 400 copies/mL); a viral load value of 399 copies/mL was assigned for samples with no RNA detected or RNA < LOQ. ARV drugs were detected using a qualitative assay that detects 22 drugs in five classes (limit of detection [LOD]: 2 ng/mL or 20 ng/mL, depending on the drug) [70].

HIV antibody profiling was performed using the VirScan assay, as described previously [47, 63]. This assay uses phage display to quantify antibody binding to a library of overlapping peptides spanning the expressed genomes of >200 viruses, including >3,300 HIV peptides representing multiple HIV subtypes and strains [47, 63]. In this assay, plasma is incubated with the phage library and antibody-bound phage are immunocaptured using beads coated with protein A and protein G. Primers with sample-specific barcodes are used to amplify the peptide-encoding DNA in immunocaptured phage; the amplified DNA is then sequenced to determine the amino acid sequences of peptides bound by the antibodies in each sample. In this study, sequencing was performed using the NovaSeq 6000 with the S2 flowcell (Illumina, San Diego, CA).

### VirScan data analysis

Each immunoprecipitation plate included 7–8 negative controls (beads only) and 3 positive controls (pooled plasma from other study participants infected >2 years with viral loads >2,000 copies/mL). Raw read counts from the VirScan assay were based on exact matching of the initial 50 nucleotides for each read. Fold change values and associated p-values were determined by comparing observed read counts to those in negative control reactions using the exact test for the negative binomial distribution in the edgeR package [71, 72]. Fold change values were adjusted by setting the value to one under the following conditions: read count <15, fold change <3, and/or p-value >0.001. Adjusted fold change values >1 represented significant antibody reactivity. VARscore values were calculated from VirScan data using the ARscore package v0.2.0 [73].

### Statistical methods

Peptide clusters with high levels of antibody reactivity at the cohort-level were identified based on having two or more peptides with cohort-level mean antibody reactivity (log_10_ fold change) >0.5 (i.e., adjusted fold change >3.16). Viral load and antibody reactivity (fold change) values were log_10_-transformed prior to statistical analysis. Analysis of associations between antibody reactivity to HIV peptides and HIV viral load was performed using simple linear regression; this analysis was limited to HIV peptides that had significant antibody reactivity (adjusted fold change >1) for one or more participants. Multiple comparisons correction was performed using two methods: a) q-values calculated from observed p-values to control the false discovery rate (where q-values <5% indicated statistical significance) [74], and b) the Bonferroni method. Epitopes in overlapping peptides associated with lower HIV viral load were identified with epitopefindr v1.1.30 [75]. Epitope logos were generated using ggseqlogo v0.1 [76]. Epitope-level reactivity was determined by selecting the maximum fold change value for any peptide containing that epitope. HIV-1 VARscore values refer to the mean VARscore value across all HIV-1 subtypes. Analysis of associations between two continuous variables was performed using simple linear regression. Between-group comparisons for categorical variables were performed using Fisher’s exact test. Between-group comparisons for continuous variables were performed using the Wilcoxon rank-sum test. Statistical analyses were performed using the statistical environment R [77]. Data were visualized using base R and ggplot2 [78].

### Informed consent

HPTN 071 participants provided written informed consent before study enrollment. HPTN 071 was approved by the institutional review boards and ethics committees of the London School of Hygiene and Tropical Medicine, the University of Zambia, and Stellenbosch University. Data and samples used for this work were accessed between 1/1/2020 and 12/312023. The authors did not have access to information that could be used to identify individual study participants.

## Results

### Study cohort

This report evaluated a subset of the 978 participants who acquired HIV infection during the HPTN 071 trial [65]. The study cohort included 77 seroconverters (13 controllers [viral load <2,000 copies/mL with no ARV drugs detected at two annual study visits], 64 non-controllers [viral load ≥2,000 copies/mL with no ARV drugs detected at two annual study visits]). VirScan testing was performed using samples collected at the second HIV-positive study visit (infection duration: 1–2 years). The mean viral load at this visit was 802 copies/mL for the controller group (interquartile range [IQR]: 399, 1,180) and 101,393 copies/mL for the non-controller group (IQR: 7,110, 80,098). There was no significant difference between groups for biological sex, age, or study country (S1 Table in S1 File).

**Fig 1:**
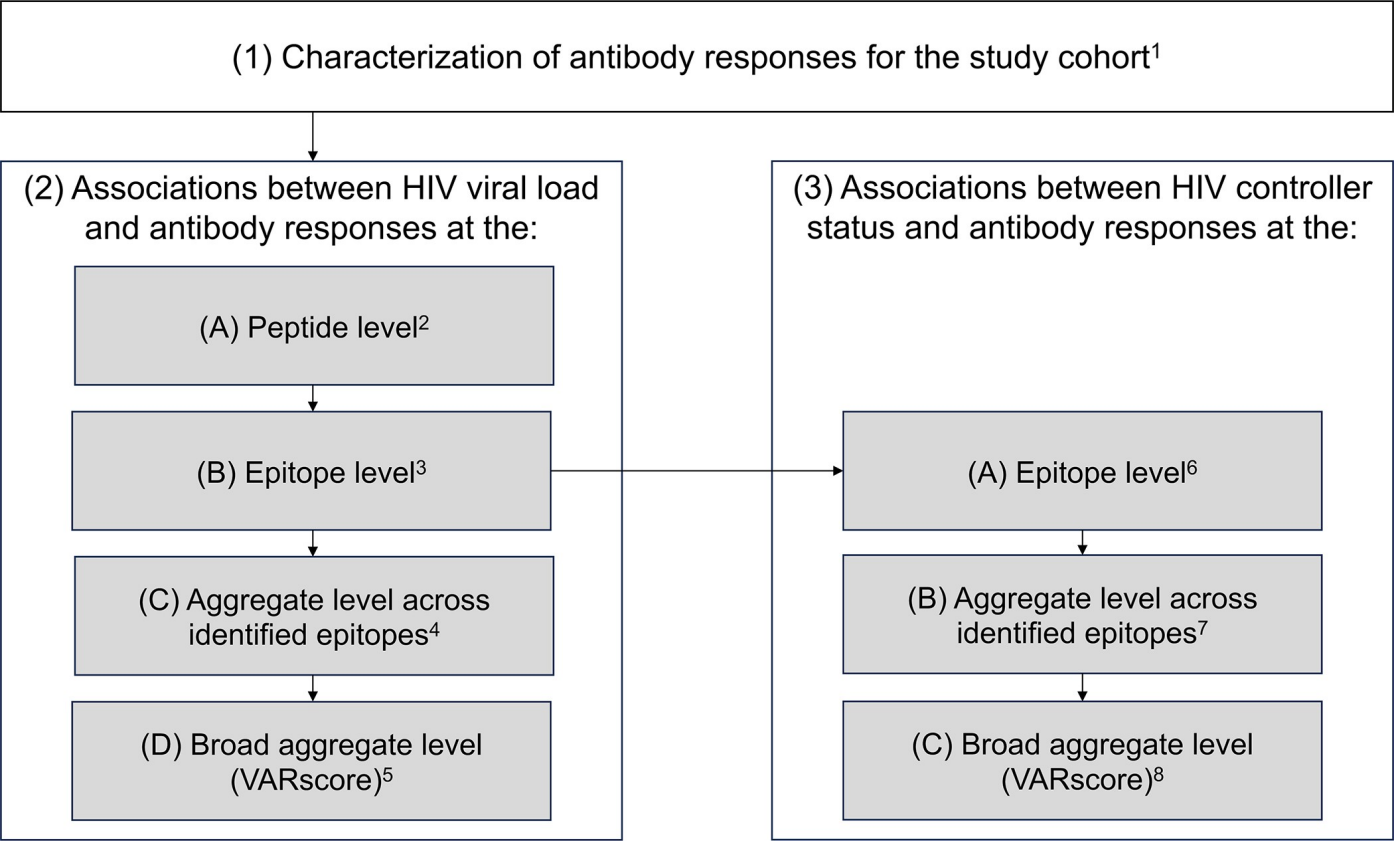
Study overview. The figure shows an overview of the assessments in this report. Antibody profiles were first characterized for the study cohort. Antibody responses were then evaluated at the peptide, epitope, and aggregate levels for associations with HIV viral load, and at the epitope and aggregate levels for associations with HIV controller status. Footnotes: ^1^ This analysis is shown in Fig 2. ^2^ “Peptide” refers to a single peptide in the VirScan library. This analysis is shown in Fig 3; the peptides associated with HIV viral load are described in S2 Table in S1 File. ^3^ “Epitope” refers to a common amino acid sequence shared by overlapping peptides. The epitopes associated with HIV viral load are described in Fig 4. ^4^ This analysis is shown in Fig 5, Panels A-B. ^5^ This analysis is shown in Fig 5, Panel C. ^6^ This analysis included the epitopes identified in step 2B and is shown in Fig 6. ^7^ This analysis is shown in Fig 7, Panels A-C. ^8^ This analysis is shown in Fig 7, Panel D and included 36 additional participants who were virally suppressed on antiretroviral treatment.

Fig 1 provides an overview of the analyses in this report. Antibody responses were first characterized for the study cohort and were then evaluated at the peptide level (reactivity to a single peptide from the VirScan library), epitope level (reactivity to a common amino acid sequence shared by overlapping peptides), and aggregate level for associations with HIV viral load and HIV controller status.

### HIV antibody reactivity in the study cohort

**Fig 2:**
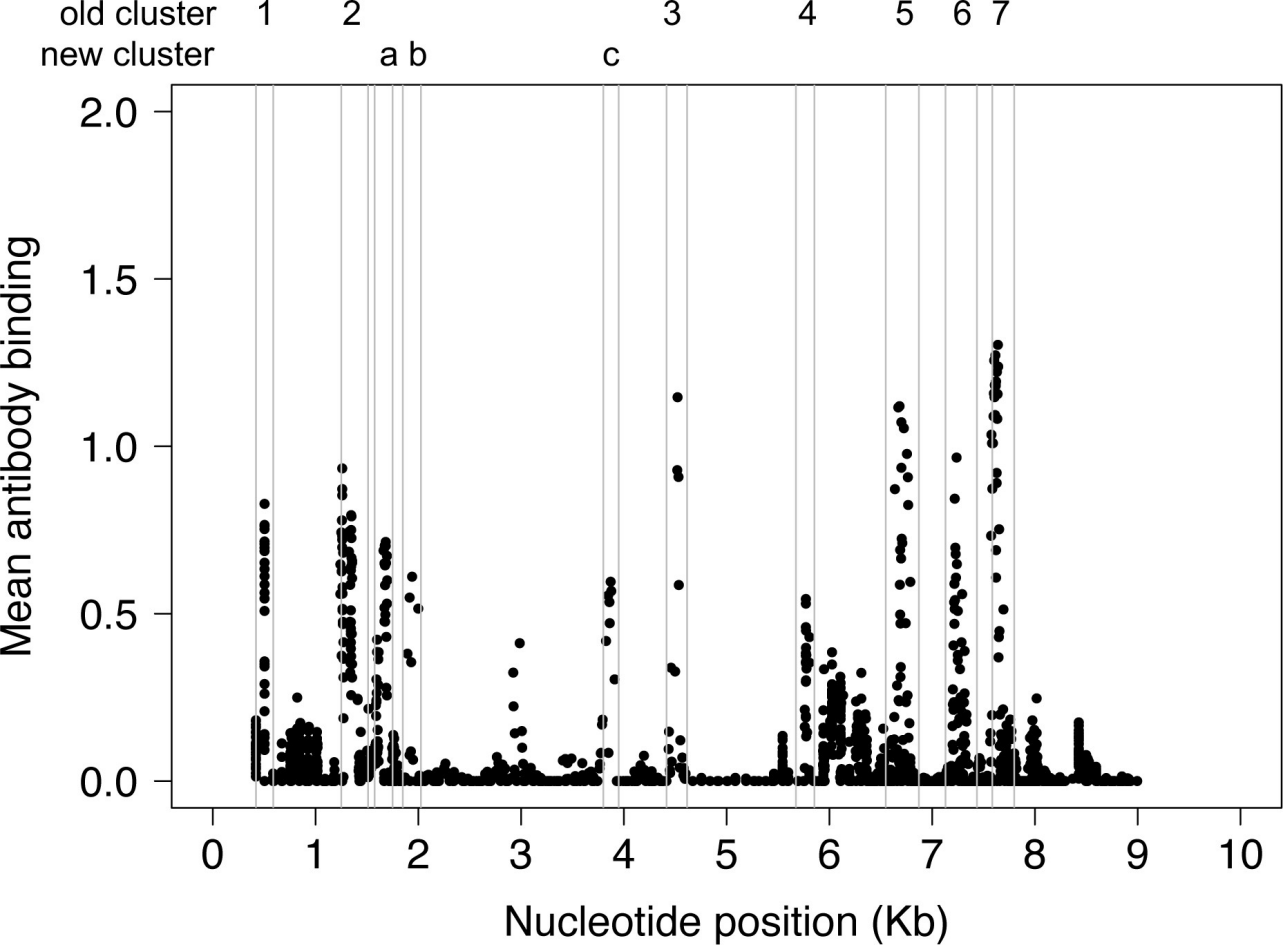
Antibody reactivity to peptides spanning the HIV genome. The plot shows the mean level of antibody binding to HIV peptides in the VirScan library for all 77 study participants analyzed one to two years after HIV infection. The x-axis shows the nucleotide position relative to genomic coordinates for the HIV HXB2 reference strain (NCBI #NC_001802). The y-axis shows mean antibody binding (log_10_ fold change); each dot represents the mean antibody binding result for one peptide. The genomic locations of ten peptide clusters with high levels of mean antibody reactivity are indicated by vertical gray lines. Seven of the peptide clusters were identified in a prior study (cluster 1: gag [p17]; cluster 2: gag [p24]; cluster 3: integrase; cluster 4: vpu; clusters 5 and 6: envelope [gp120]; cluster 7: envelope [gp41]) [64]. Three new peptide clusters were identified in this study (cluster a: gag [p7]; cluster b: protease; cluster c: integrase). Abbreviations: Kb: kilobase.

VirScan was used to characterize antibody responses to HIV peptides in the 77 study participants (Fig 2). Ten clusters of HIV peptides had high levels of antibody reactivity (defined as having two or more peptides with mean antibody reactivity [log_10_ fold change] >0.5). Seven of these clusters were identified in a prior report that evaluated antibody responses among HIV controllers and non-controllers with unknown duration of infection (cluster 1: gag [p17; N-terminus]; cluster 2: gag [p24; C-terminus]; cluster 3: integrase [C-terminus]; cluster 4: vpu [N-terminus]; cluster 5: envelope [gp120; V3 loop and CD4 binding loop]; cluster 6: envelope [gp120/gp41; V5 and fusion peptide]; cluster 7: envelope [gp41; C-terminal heptad repeat region, HR2]) [64]. The three additional peptide clusters identified in this report had high levels of antibody reactivity to the following targets: cluster a: the first zinc finger region of the nucleocapsid protein, gag p7; cluster b: the N-terminus of protease, including the active site; and cluster c: the N-terminus of integrase.

### Associations between antibody reactivity and HIV viral load

#### Peptide-level responses

**Fig 3:**
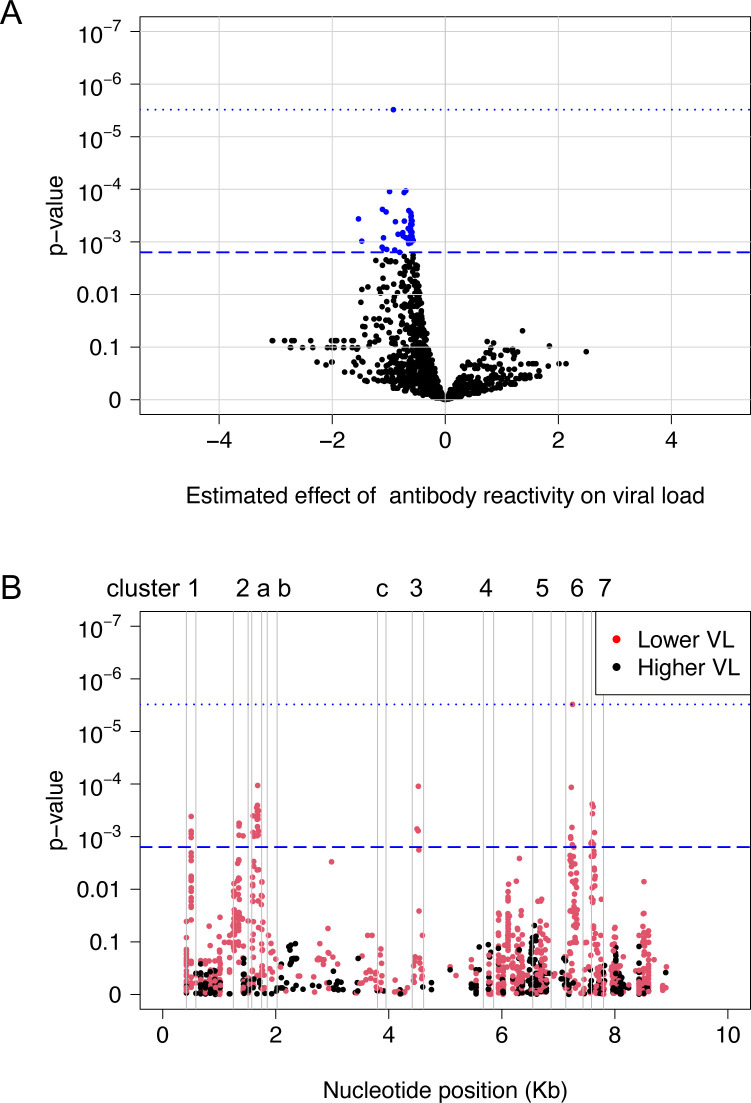
Peptide-level antibody responses and HIV viral load. The plots show the association between the level of antibody reactivity to HIV peptides and HIV viral load as determined by linear regression. Data are shown for the 77 participants in the study cohort; this analysis included 1,235 HIV peptides that had significant antibody reactivity (adjusted fold change >1) for at least one participant. Panel A: The volcano plot shows the significance of the association between the level of antibody reactivity and viral load. The x-axis shows the estimated effect of antibody reactivity on viral load (estimated effect from the linear regression). Positive values indicate that higher levels of antibody reactivity were associated with higher viral loads; negative values indicate that higher levels of antibody reactivity were associated with lower viral loads. The y-axis shows the -log_10_ p-value for the association between the level of antibody reactivity and viral load. Each dot represents data for a single peptide; blue dots indicate peptides with a significant association. The blue dashed line indicates the highest q-value <5% (q = 0.0453); this corresponds to a p-value of 0.00158. The dotted blue line indicates the cutoff for significance using the Bonferroni correction (p = 0.05/1,235 = 4.0 x 10^−5^). Panel B: The plot shows the same data and significance thresholds visualized across the viral genome. The x-axis shows nucleotide position relative to genomic coordinates for the HIV HXB2 reference strain (NCBI #NC_001802). The y-axis shows the -log_10_ p-value for the association between antibody reactivity and viral load. Black dots indicate peptides for which higher antibody reactivity was associated with higher viral loads; red dots indicate peptides for which higher antibody reactivity was associated with lower viral loads. The genomic locations of the ten peptide clusters from Fig 2 are indicated by vertical gray lines. Abbreviations: Kb: kilobase; VL: viral load.

We next evaluated whether antibody reactivity to individual peptides was associated with HIV viral load (Fig 3). This analysis included the 1,235 HIV peptides with significant antibody reactivity (adjusted fold change >1) that were detected in samples from one or more participants. Antibody reactivity to 43 peptides was significantly associated with viral load after multiple testing correction to control for the false discovery rate (q<0.05, p≤0.00158). Using the Bonferroni correction method, antibody reactivity to one peptide remained significantly associated with viral load (p = 3.1 x 10^−6^; Peptide ID: 18306). For all 43 peptides, higher levels of antibody reactivity were associated with lower viral loads.

The 43 peptides were located in the six clusters of homologous peptides that had high levels of mean antibody reactivity for the cohort (Fig 1, S2 Table in S1 File). Twenty-six peptides were located in gag (four in the N-terminal region of p17 [cluster 1], five in the C-terminal region of p24 [cluster 2] and 17 in the C-terminal region of p7 [cluster a]). Three peptides were located in the C-terminal region of integrase (cluster 3). The remaining 14 peptides were located in env (seven in the region spanning the V5 loop of gp120 and fusion peptide of gp41 [cluster 6] and seven in the HR2 of gp41 [cluster 7]).

#### Epitope-level responses

**Fig 4:**
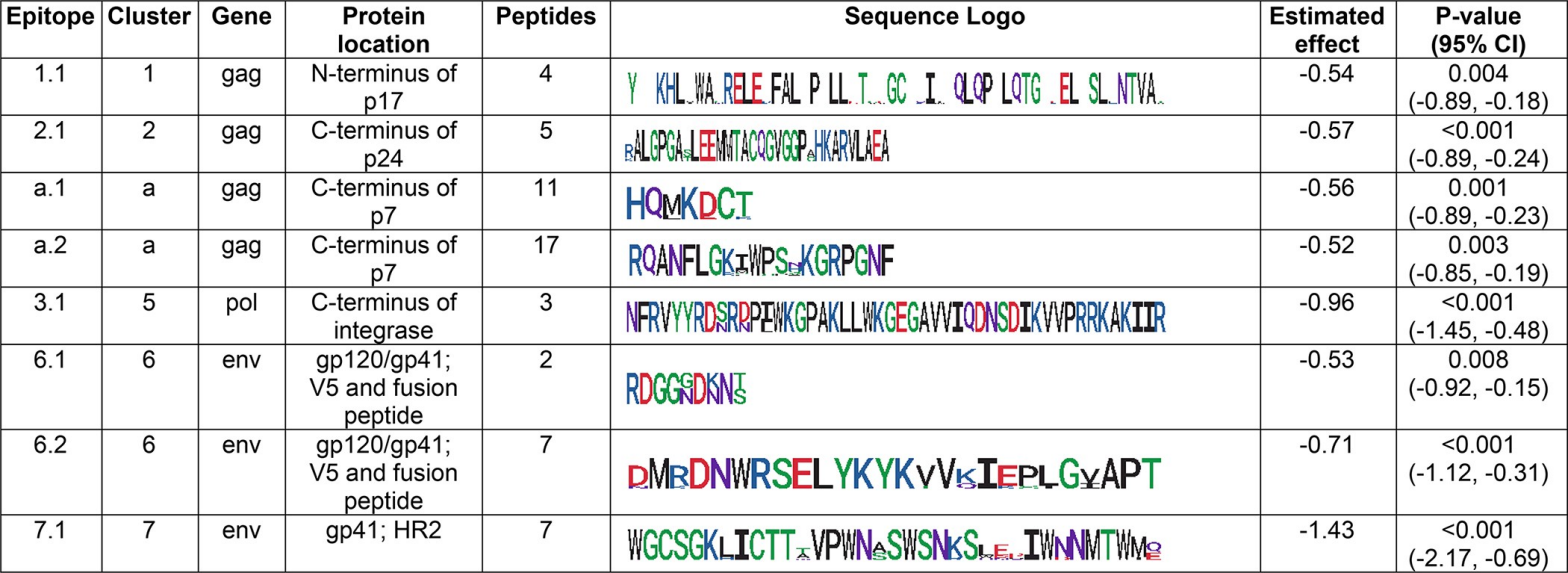
HIV antibody epitopes associated with lower HIV viral load.

The program eptiopefindr [75] was used to identify common epitopes for the peptides in each cluster (Fig 4). The number of peptides with each epitope ranged from two to 17. Clusters 1, 2, 3, and 7 each contained one common epitope shared by peptides in the cluster, while Clusters a and 6 each contained two common epitopes. The association between antibody reactivity and HIV viral load remained statistically significant when the analysis was performed at the epitope level for each of the eight epitopes. Estimated effect for the association ranged from -1.430 to -0.520; this measure indicates the change in viral load (log_10_ scale) associated with one unit increase in antibody reactivity (log_10_ scale) (i.e., if the estimated effect were -0.5, then when comparing two participants that differ tenfold in antibody reactivity, we would expect the participant with higher reactivity to have a 32% [10^−0.5^] lower viral load).

The figure shows the features of the eight epitopes where higher antibody reactivity was associated with lower viral load. These epitopes were identified from 43 peptides located in six clusters (Fig 3; S2 Table in S1 File). HIV gene and protein locations were determined based on full-length peptides. Sequence logos were generated using ggseqlogo v0.1 [76]. Estimated effect and associated p-values were calculated using simple linear regression between antibody reactivity and viral load. The estimated effect indicates the change in viral load (log_10_ scale) associated with a unit increase in antibody reactivity (log_10_ scale). Negative values indicate that a unit increase in antibody reactivity was associated with a decrease in viral load.

Abbreviations: gp: glycoprotein; HR: helical region; 95% CI: 95% confidence intervals.

### Aggregate responses

**Fig 5:**
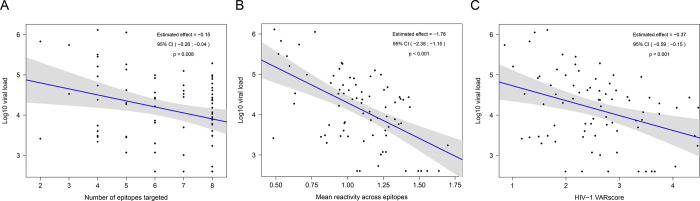
Aggregate antibody responses and HIV viral load. The plots show the association between three aggregate measures of HIV antibody reactivity and HIV viral load, as determined by linear regression. Data are shown for the 77 participants in the study cohort. For each panel, each dot represents data for a single participant. The y-axes show the HIV viral load (log_10_ scale). The blue lines indicate the least squares regression lines. P-values indicate the significance of the associations as determined by linear regression. Grey regions show the 95% confidence bands for the mean antibody response.

We next evaluated whether aggregate antibody reactivity to the epitopes described in Fig 4 was associated with HIV viral load (Fig 5). We found a significant association between the total number of epitopes targeted and HIV viral load (estimated effect: -0.15, 95% CI: -0.26, -0.04, p = 0.008); here, estimated effect indicates the change in viral load (log_10_ scale) associated with one additional targeted epitope. There was also a significant association between participant mean antibody reactivity (fold change) across all eight epitopes and HIV viral load (estimated effect: -1.76, 95% CI: -2.38, -1.15, p<0.001); here, estimated effect indicates the change in viral load (log_10_ scale) associated with one unit increase in mean antibody reactivity (log_10_ scale).

Panel A: Aggregate antibody reactivity was evaluated for the eight HIV epitopes shown in Fig 4. The x-axis shows the number of epitopes targeted (adjusted fold change >1). The estimated effect indicates the change in viral load (log_10_ scale) associated with one additional targeted epitope. Panel B: Mean antibody reactivity was evaluated across all eight HIV epitopes shown in Fig 4. The x-axis shows the mean antibody reactivity (log_10_ fold change) across all eight epitopes. The estimated effect indicates the change in viral load (log_10_ scale) associated with one unit increase in mean antibody reactivity (log_10_ scale). Panel C: The VARscore is a composite measure of the overall breadth and strength of antibody reactivity to all peptide targets across a viral genome, as measured by VirScan. The x-axis shows the HIV-1 VARscore. The estimated effect indicates the change in viral load (log_10_ scale) associated with one unit increase in HIV-1 VARscore.

The VARscore is a composite value that combines VirScan data for all peptide targets across a viral genome; this provides an aggregate measure of the overall breadth and strength of antibody reactivity to a virus [73]. We next evaluated whether HIV-1 VARscore was associated with HIV viral load (Fig 5). There was a significant association between HIV-1 VARscore and HIV viral load (estimated effect: -0.37, 95% CI: -0.59, -0.15, p = 0.001); here, estimated effect indicates the change in viral load (log_10_ scale) associated with one unit increase in HIV-1 VARscore.

#### Associations in the non-controller participant subset

We next evaluated whether the associations between antibody reactivity and HIV viral load were still observed when data from the 13 controllers were removed from the analysis. At the peptide level, we analyzed the 1,183 HIV peptides that had significant antibody reactivity in samples from one or more of the 64 non-controllers (S1 Fig in S1 File); this analysis did not identify any peptides where antibody reactivity was significantly associated with viral load after multiple testing correction. At the epitope-level, associations between antibody reactivity and viral load were still observed when the 13 controllers were excluded (S3 Table in S1 File). The association remained statistically significant for five of the eight epitopes (epitopes 2.1, 3.1, 6.1, 6.2, and 7.1; estimated effect range: -0.977 to -0.352); the association between epitope-level antibody reactivity to the other three epitopes (1.1, a.1 and a.2) and viral load was not significant.

As a final step in this portion of the analysis, we evaluated whether aggregate antibody reactivity was associated with viral load when the 13 controllers were excluded (S2 Fig in S1 File). In this analysis, the association between the number of epitopes targeted (adjusted fold change >1) and viral load was not significant (estimated effect: -0.08, 95% CI: -0.17, 0.01, p = 0.094); here, estimated effect indicates the change in viral load (log_10_ scale) associated with one additional targeted epitope. In contrast, we still observed a significant association between participant mean antibody reactivity (fold change) across all eight epitopes and viral load for the non-controller group (estimated effect: -1.17, 95% CI; -1.79, -0.54; p<0.001); here, estimated effect indicates the change in viral load (log_10_ scale) associated with one unit increase in mean antibody reactivity (log_10_ scale). The association between HIV-1 VARscore and viral load also remained significant for the non-controller subset (estimated effect: -0.24, 95% CI: -0.44, -0.05; p = 0.016); here, estimated effect indicates the change in viral load (log_10_ scale) associated with one unit increase in HIV-1 VARscore.

### Associations between antibody reactivity and HIV controller status

#### Epitope-level responses

**Fig 6:**
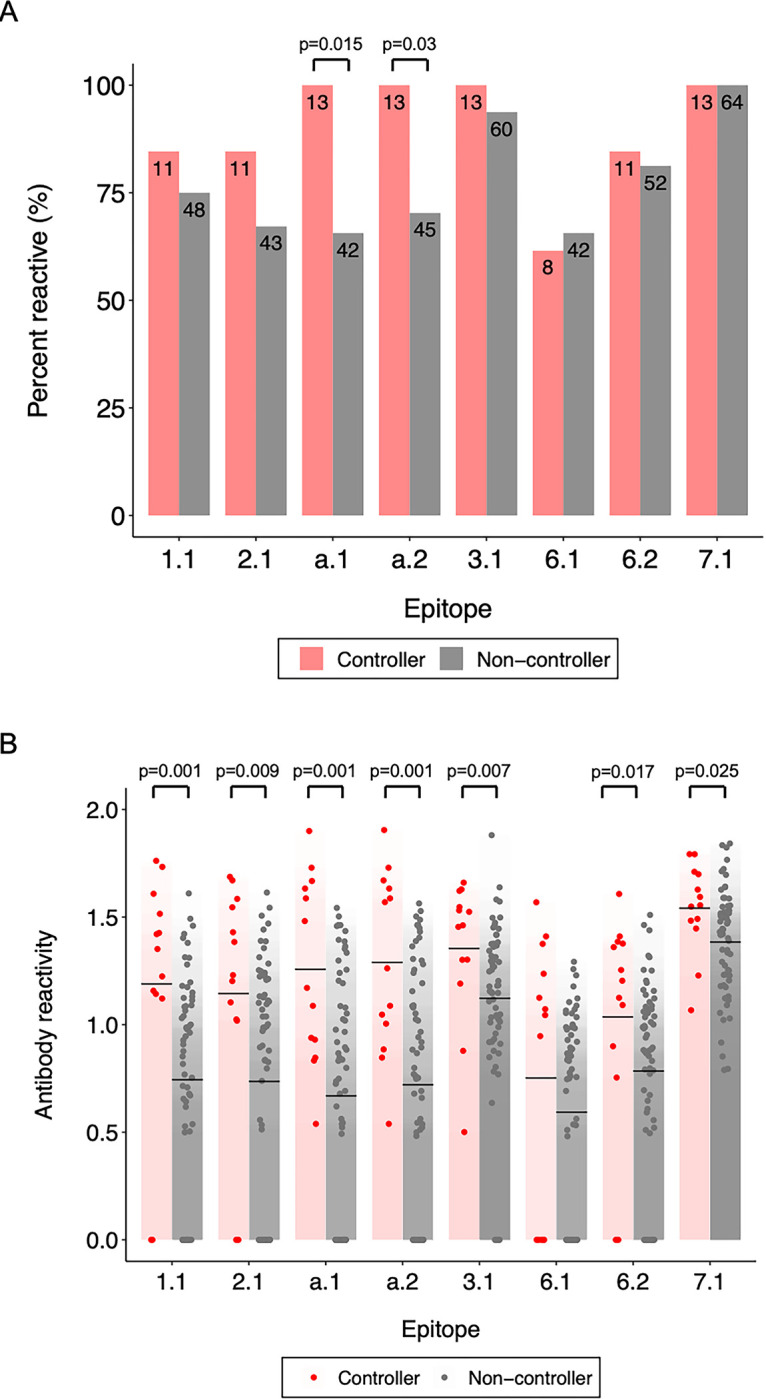
Epitope-level antibody responses in controllers vs. non-controllers. Antibody reactivity was assessed for the HIV epitopes shown in Fig 4 for two participant groups: controllers (n = 13; red) and non-controllers (n = 64; grey). Panel A: The plot shows the frequency of reactivity to each epitope in each group (reactive: adjusted fold change >1; not reactive: adjusted fold change = 1). P-values show the significance of the association between controller status and the prevalence of reactivity using Fisher’s exact test. Panel B: The plot shows antibody reactivity (log_10_ fold change) to each epitope; each dot indicates data for one participant. Mean values for each group are indicated by black crossbars. P-values show the significance of the association between controller status and the level antibody reactivity based on Wilcoxon rank-sum test statistics.

We next compared antibody reactivity to each of the eight epitopes in controllers (n = 13) vs. non-controllers (n = 64) (Fig 6). Panel A shows the frequency of antibody reactivity (adjusted fold change >1) to each of the epitopes in the two groups. Antibody reactivity to two epitopes was observed more frequently among controllers than non-controllers (epitope a.1: 13/13 [100.0%] vs. 42/64 [65.6%], p = 0.015; epitope a.2: 13/13 [100.0%] vs. 45/64 [70.3%], p = 0.03); for the remaining epitopes, there was no significant difference in the prevalence of antibody reactivity between groups. Panel B shows mean antibody reactivity (fold change) to each of the eight epitopes in the two groups. Mean antibody reactivity to seven epitopes was higher among controllers than non-controllers (epitope 1.1: 24.7 vs. 9.3, p = 0.001; epitope 2.1: 22.2 vs. 10.3, p = 0.009; epitope a.1: 27.2 vs. 9.2, p = 0.001; epitope a.2: 28.4 vs. 10.1, p = 0.001; epitope 3.1: 27.4 vs. 17.4, p = 0.007; epitope 6.2: 16.4 vs 9.0, p = 0.017; epitope 7.1: 38.2 vs. 28.1, p = 0.025); there was no significant difference in mean antibody reactivity to epitopes 6.1 between the two groups (which may be due to low power; epitope 6.1. only had two peptides, the lowest among all epitopes).

#### Aggregate responses

**Fig 7:**
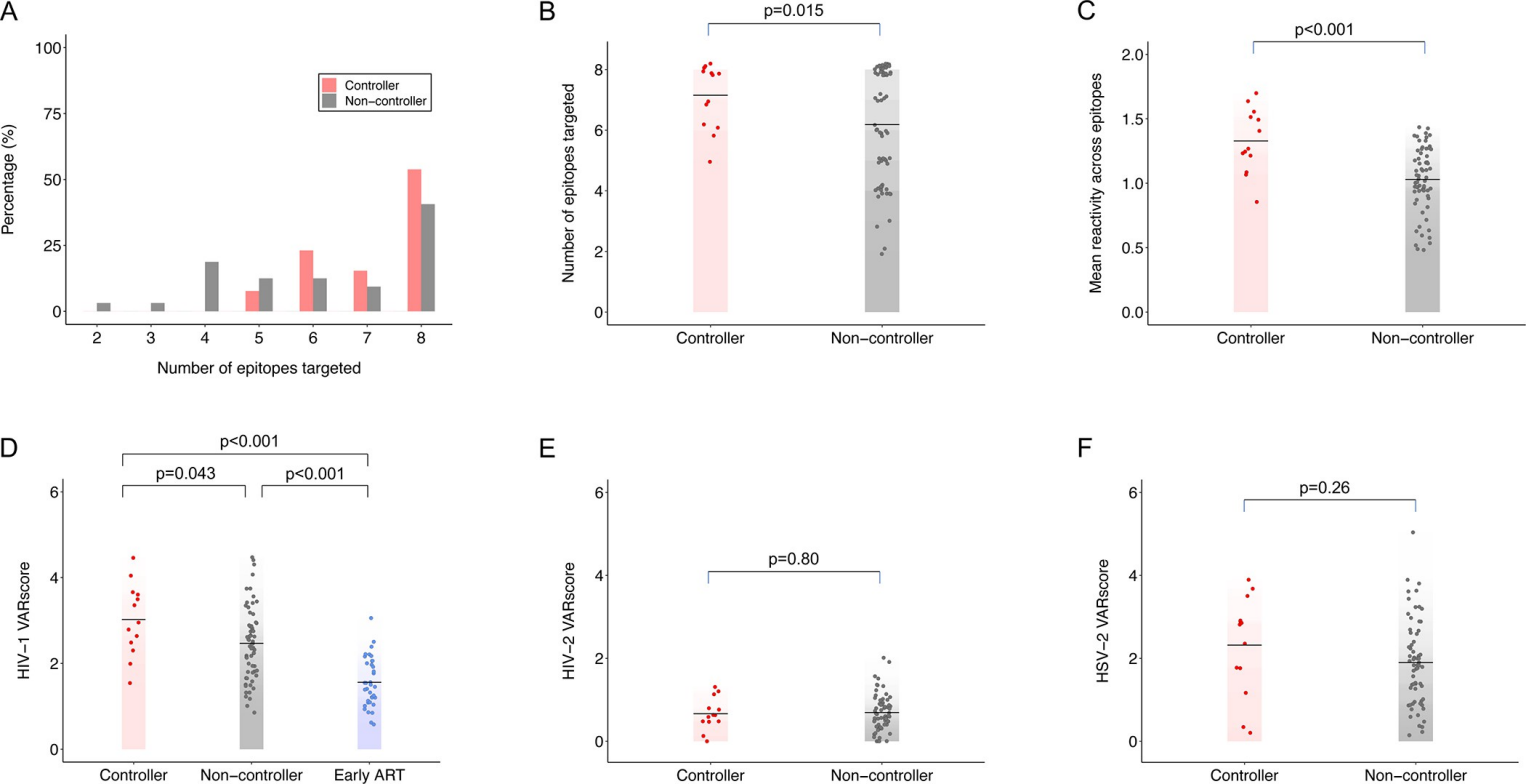
Aggregate antibody responses in controllers vs. non-controllers. Aggregate antibody reactivity was assessed for controllers (n = 13; red) and non-controllers (n = 64; grey). Panel A: Aggregate antibody reactivity was evaluated for the eight HIV epitopes shown in Fig 4. The histogram shows the number of epitopes targeted by participants based on controller status. Data were binned according to the number of epitopes targeted by each study participant. Bar heights indicate frequency. Panel B: The plot shows the number of epitopes targeted based on controller status; each dot indicates the number of epitopes targeted for one study participant. Mean values for each group are indicated by black crossbars. P-values show the significance of the association between controller status and antibody reactivity based on t-statistics. Panel C: The plot shows the mean antibody reactivity (mean log_10_ fold change) across all selected epitopes based on controller status; each dot indicates mean data for one study participant. Mean values for each group are indicated by black crossbars. P-values show the significance of the association between controller status and antibody reactivity based on t-statistics. Panel D: The VARscore is a composite measure of the overall breadth and strength of antibody reactivity to all peptide targets across a viral genome, as measured by VirScan. The plot shows HIV-1 VARscores for controllers (N = 13, red) and viremic non-controllers (N = 64, grey); this analysis also included a group of non-controllers who were suppressed on antiretroviral therapy within the first year of HIV infection (N = 36, blue; see Methods). Each dot indicates HIV-1 VARscore data for one study participant. Mean values for each group are indicated by black crossbars. P-values show the significance of the association between controller status and HIV-1 VARscore based on t-statistics. Panels E-F: The plots show VARscores for HIV-2 (Panel E) and HSV-2 (Panel F) for controllers (n = 13; red) vs. non-controllers (n = 64, grey). Each dot indicates data for one participant. Mean values for each group are indicated by black crossbars. P-values show the significance of the association between controller status and the VARscore based on t-statistics.

We next compared aggregate antibody reactivity to the eight epitopes for controllers vs. non-controllers (Fig 7, Panels A-C). The number of epitopes targeted ranged from five to eight for controllers and from two to eight for non-controllers. The mean number of epitopes targeted was higher among controllers vs. non-controllers (7.15 vs. 6.19, p = 0.015). The participant mean antibody reactivity (fold change) across all eight epitopes was also higher for controllers vs. non-controllers (21.31 vs. 10.70, p<0.001). Both measures indicate that controllers reacted more broadly across the eight epitopes than non-controllers.

We then compared HIV-1 VARscores for controllers vs. non-controllers (Fig 7, Panel D); this analysis included an additional group of 36 participants who were virally suppressed on ART (viral load <400 copies/mL with ARV drugs detected at both HIV-positive study visits). Mean HIV-1 VARscores were higher for controllers than non-controllers (3.03 vs. 2.47, p = 0.043), indicating that controllers had stronger overall HIV-1 specific antibody responses than non-controllers. Mean HIV-1 VARscores were lower for participants suppressed on ART than controllers (1.56 vs. 3.03, p<0.001) and non-controllers (1.56 vs. 2.47, p<0.001); this finding is consistent with prior studies that demonstrate a down-regulation of HIV antibody expression in persons who are virally suppressed on ART [47–50].

As a final step, we compared VARscores for two other viruses to assess whether the findings in Fig 6 were specific for HIV-1. This analysis was performed for HIV-2, which was expected to be uncommon in this cohort, and HSV-2, which was expected to be highly prevalent in this cohort (Fig 7). For both viruses, mean VARscores were similar for controllers and non-controllers (HIV-2: 0.66 vs. 0.69, p = 0.80; HSV-2: 2.32 vs. 1.90, p = 0.25). This indicates that the observed differences in HIV-1 VARscores were HIV-1 virus-specific and did not reflect general differences in the breadth and strength of the antibody response in controllers vs. non-controllers.

## Discussion

In this report, we used VirScan to characterize HIV antibody responses associated with viral load and controller status among persons who had been living with HIV for one to two years. These persons were enrolled in a community-randomized trial that recruited participants from the general population in Zambia and South Africa. We identified ten peptide clusters that served as the primary targets of HIV antibodies in this cohort (three in env, three in gag, two in integrase, and one each in protease and vpu). Seven of these clusters (clusters 1–7) overlapped with clusters identified in our previous study [64]. This was consistent with the findings from our earlier report in an independent cohort with a different prevalent HIV subtype (prior study: subtype B; current study: subtype C). Three new peptide clusters (clusters a-c) were also identified in this report. The new clusters could represent epitopes that are more commonly targeted in subtype C HIV. High-level reactivity to these targets could also be more common in the first 1–2 years of HIV infection [47] or could reflect other differences in the cohorts used for analysis in this report and our prior report [64].

We found that higher levels of antibody reactivity to 43 HIV peptides representing 8 unique epitopes were associated with lower HIV viral loads. All eight epitopes were located in the clusters commonly targeted by both controllers and non-controllers, suggesting that more robust antibody responses to standard HIV targets, rather than responses to unique targets, may play a role in controlling viral replication. HIV controllers reacted more frequently to two of these epitopes (a.1 and a.2) and had higher mean antibody reactivity to seven of these epitopes (1.1, 2.1, a.1, a.2, 3.1, 6.2, and 7.1). Three of these seven epitopes and 26 (72.2%) of the 36 corresponding peptides are located in gag. These findings are consistent with prior studies that found robust controller antibody responses to broad gag targets [55, 60–62].

When antibody reactivity to all eight HIV epitopes was assessed as a composite measure, both the number of epitopes targeted and the mean reactivity across the eight epitopes was associated with lower viral load. Higher reactivity to targets across the HIV genome (HIV-1 VARscore [73]) was also associated with lower viral load. These associations remained significant when we compared reactivity in controllers and non-controllers. HIV controllers targeted more of the eight epitopes, had higher mean reactivity across all eight targets, and had significantly higher mean HIV-1 VARscores than non-controllers. These findings are consistent with general differences in the breadth of the antibody response that we observed in our prior study of controllers vs. non-controllers with unknown duration of infection [64]. Taken together, our findings suggest that broad, robust antibody responses to standard HIV targets may contribute to viral containment and HIV controller status.

In this study, 7/13 (54%) of the controllers had a viral load below the limit of quantification (400 copies/mL) and were assigned a viral load value of 399 copies/mL. Using this conservative approach, we identified 43 peptides where the level of antibody reactivity was significantly associated with viral load; for all of these peptides, higher levels of antibody reactivity were associated with lower viral loads (Fig 3). Using the largest possible value below the limit of quantification for "censored" participants assured that the type I error was actually an upper bound and that we could be confident in the significance of the association with viral load. Since this approach might increase the number of false negative results, we conducted additional sensitivity analyses. When we used an assigned value of 200 copies/mL or more, we did not observe large numbers of additional peptides showing significance. Only when the imputed vial load value was consistently below 200 copies/mL for each of the seven censored participants did we observe a somewhat larger increase in the number of significant peptides. In all simulation scenarios, the 43 peptides remained significantly associated with viral load.

To our knowledge, none of the bnAbs currently under investigation for HIV treatment and prevention target epitopes located in the same regions of the corresponding HIV proteins as the peptides identified in this study [79–81]. Notably, one of these epitopes (7.1) overlaps with an HR2 epitope that we previously demonstrated was preferentially targeted prior to infection in persons who were able to control infection after HIV acquisition [68]. We did not identify any peptides or epitopes where higher levels of antibody reactivity were associated with higher HIV viral load or non-controller status. This was unexpected, since viral suppression from ART generally leads to a reduction in antibody titer due to reduced antigen exposure [47–50], which was consistent with our findings of lower HIV-1 VARscores in persons on ART as compared to both controllers and non-controllers.

Viral suppression on ART can improve health outcomes for PWH and reduce risk of HIV-related mortality [8–15]. HPTN 071 and global health programs have also demonstrated that reducing viral load at the community level with “universal testing and treatment” strategies can significantly reduce HIV incidence [66, 82]. These findings led UNAIDS to establish “95-95-95” Fast-Track targets based on mathematical models indicating that achieving 95% success in each step of the HIV care cascade (diagnosis, linkage to care, viral suppression on ART) would effectively curb the epidemic [83]. Unfortunately, significant structural barriers to universal ART delivery still remain in some resource-limited settings [84, 85].

Significant reductions in HIV incidence may still be achieved with more modest levels of community-wide viral load reduction. A modeling study found that lower viral loads in North America vs. sub-Saharan Africa (difference of ~0.5 log_10_ viral load) may significantly contribute to observed geographic differences in HIV incidence [86]. Other studies have demonstrated that similar reductions in population-level viral load were associated with reduced HIV incidence [87–89]. The findings in this report suggest that enhancing the depth and breadth of HIV antibody responses (potentially with pre-infection or therapeutic vaccination [90–93]) could help lower community-level viral load and reduce HIV incidence. This approach may offer advantages in settings with barriers to universal ART delivery. Further research could evaluate whether the epitopes identified in this report might be useful targets for immune-based interventions for modulating HIV viral load.

This study has several limitations. First, despite the large size of the HPTN 071 trial (>48,000 persons enrolled and followed), we were only able to identify 13 controllers with known duration of infection. Second, the HPTN 071 cohort only included participants from Zambia and South Africa, where the vast majority of infections are caused by subtype C infection HIV; the HPTN 071 cohort also included a disproportionate number of women (74%). These factors may limit the generalizability of our findings. Third, the viral load assay that was used in HPTN 071 had a LOQ of <400 copies/mL [66]; the plasma samples stored in this trial did not have sufficient volume for testing with a more sensitive viral load assay. For this reason, we were not able to evaluate factors associated with elite control of HIV infection. Fourth, the VirScan assay measures IgG binding to unglycosylated, linear epitopes; therefore, we were not able to assess reactivity for other antibody isotypes or against glycosylated or conformational epitopes. Fifth, the measure of antibody reactivity provided by the VirScan assay reflects both antibody titer and avidity; therefore, we were not able to assess whether the observed associations between antibody reactivity, viral load, and controller status were driven by differences in antibody titer, antibody avidity, or a combination of both factors. Sixth, CD4 cell count data was not collected in HPTN 071, cellular samples were not stored, and consent was not obtained for host genetic testing; therefore, we were not able to evaluate the association of viral load and HIV control with other factors, such as host HLA type [69] and cellular immune responses [94–97]). Seventh, the viral loads were too low in most controllers for HIV genotyping; this limited our ability to evaluate viral factors associated with viral load and controller status [98, 99]. Eighth, we assessed antibody profiles at a single timepoint (infection duration: 1–2 years); further research in cohorts with known duration and longer post-infection follow-up could be used to evaluate the evolution of these responses and their association with viral load over the full HIV disease course. Finally, it is possible that the higher levels of antibody reactivity that we observed in persons with lower viral loads could be a consequence of HIV control (rather than the cause), reflecting more robust immune systems among those with a greater capacity for viral containment. If the findings from this study are confirmed in other cohorts, further studies could be performed to determine whether enhancing reactivity to the HIV epitopes identified in this study (e.g., with vaccination or passive immunization) results in a reduction in HIV viral load.

## Conclusion

We identified HIV antibody targets that are associated with lower viral load and HIV controller status one to two years after infection. We also demonstrated that robust aggregate responses to these targets and broad antibody reactivity across the HIV genome were associated with these outcomes. These findings provide novel insights into the relationship between humoral immunity and viral containment, which could help inform the design of antibody-based approaches for HIV treatment and prevention.

## Supporting information

S1 File (DOCX)
