# Supplementary material for: Identification of antibody targets associated with lower HIV viral load and viremic control
Source: PLoS One. 2024 Sep 17;19(9):e0305976. doi: 10.1371/journal.pone.0305976 (PMC11407625; doi:10.1371/journal.pone.0305976)
Supplement: S1 File — (DOCX) [file pone.0305976.s001.docx]

**Supplementary Material**

Identification of antibody targets associated with lower HIV viral load and viremic control

Wendy Grant-McAuley, William R. Morgenlander, Ingo Ruczinski, Kai Kammers, Oliver Laeyendecker, Sarah E. Hudelson, Manjusha Thakar, Estelle Piwowar-Manning, William Clarke, Autumn Breaud, Helen Ayles, Peter Bock, Ayana Moore, Barry Kosloff, Kwame Shanaube, Sue-Ann Meehan, Anneen van Deventer, Sarah Fidler, Richard Hayes, H. Benjamin Larman, Susan H. Eshleman, for the HPTN 071 (PopART) Study Team

**HPTN 071 (PopART) Study Team members**

London School of Hygeine and Tropical Medicine (LSHTM) (London, UK)

Sian Floyd, Peter Godfrey-Faussett, James Hargreaves, Richard Hayes, Kalpana Sabapathy, Albertus Schaap, Deborah Watson-Jones

Imperial College (IC) (London, UK)

Sarah Fidler, Christophe Fraser, Katharina Hauck, Peter Smith

Desmond Tutu TB Center, Stellenbosch University (DTTC) (Western Cape, South Africa)

Nulda Beyers, Peter Bock, Lyn Horn

ZAMBART Project (Lusaka, Zambia)

Helen Ayles, Virginia Bond, Nathaniel Chishinga Kwame Shanaube

Division of AIDS (DAIDS) at the U.S. National Institutes of Health (NIH) (Bethesda, MD, USA)

David Burns

Vanderbilt University, Institute of Global Health (Nashville, TN, USA)

Sten Vermund

Harvard University (Boston, MA, USA)

Mark Barnes

HPTN Data Management Center (SCHARP) (Seattle, WA, USA)

Corey Kelly, Deborah Donnell, Lynda Emel, Megan Baldwin

HPTN Laboratory Center (Johns Hopkins University) (Baltimore, MD, USA)

Vanessa Cummings, Susan Eshleman, Estelle Piwowar-Manning, Shauna Wolf

HPTN Operations Center (FHI360) (Durham, NC, USA)

Sam Griffith, Ayana Moore, Nirupama Sista, Rhonda White

**Supplemental Table 1. Study cohort.**

|  |  | **Total**  (n=77) | **Controllers** (n=13)^1^ | **Non-controllers** (n=64)^2^ | **p-value** |
| --- | --- | --- | --- | --- | --- |
| **Viral load**^3^ |  |  |  |  | ND^4^ |
|  | Mean  (IQR) | 84,410  (3,070, 54,140) | 802  (399, 1,180) | 101,393  (7,110, 80,098) |  |
| **Sex** |  |  |  |  | 0.495 |
|  | Female | 57 (74.0%) | 11 (84.6%) | 46 (71.9%) |  |
|  | Male | 20 (26.0%) | 2 (15.4%) | 18 (28.1%) |  |
| **Age** |  |  |  |  | 0.500 |
|  | 18-24 | 19 (24.7%) | 2 (15.4%) | 17 (26.6%) |  |
|  | 25+ | 58 (75.3%) | 11 (84.6%) | 47 (73.4%) |  |
| **Country** |  |  |  |  | 1.000 |
|  | South Africa | 22 (28.6%) | 4 (30.8%) | 18 (28.1%) |  |
|  | Zambia | 55 (71.4%) | 9 (69.2%) | 46 (71.9%) |  |

Legend for Supplemental Table 1. The table shows viral load and demographic data for the study cohort and for each study group (controllers, non-controllers).

Footnotes:

^1^ Participants classified as controllers had viral loads <2,000 copies/mL with no ARV drugs detected at two annual study visits.

^2^ Participants classified as non-controllers had viral loads $\geq$2,000 copies/mL with no ARV drugs detected at two annual study visits.

^3^ Viral load data (HIV RNA copies/mL) are shown for the second HIV-positive study visit (infection duration 1-2 years).

^4^ This P-value was not determined because viral load was used to identify controllers.

Abbreviations: ARV: antiretroviral; IQR: interquartile range; ND: not determined.

**Supplemental Table 2. Characteristics of peptides where higher antibody reactivity was associated with lower viral load.**

| **Peptide ID** | **HIV gene location** | **HIV protein location** | **HXB2 coordinates** | **UniProt ID** | **Cluster** | **Epitope** | **Peptide sequence** |
| --- | --- | --- | --- | --- | --- | --- | --- |
| 17419 | gag | p17 | 420-588 | O12157 | 1 | 1.1 | YMMKHLVWASRELERFALDPGLLETSEGCKQIMKQLQPALQTGTKELISLHNTVAT |
| 64581 | gag | p17 | 420-588 | Q75001 | 1 | 1.1 | YMLKHLVWANRELEKFALNPDLLDTSAGCKQIIKQLQPALQTGTEELKSLFNTVAT |
| 77406 | gag | p17 | 420-588 | Q9QC00 | 1 | 1.1 | YRLKHLVWASRELERFALNPNLLETVEGCRQIIRQLQPSLQTGSEELRSLFNTVAT |
| 78255 | gag | p17 | 420-588 | Q9QSR3 | 1 | 1.1 | YKMKHLIWASRELERFALDPGLLETSEGCQKIIRQLQPSLQTGSEELKSLFNTVAV |
| 17429 | gag | p24 | 1266-1434 | O12157 | 2 | 2.1 | QDVKNWMTDTLLVQNANPDCKTILRALGPGASLEEMMTACQGVGGPGHKARVLAEA |
| 64591 | gag | p24 | 1266-1434 | Q75001 | 2 | 2.1 | QDVKNWMTDTLLVQNANPDCKTILRALGPGASLEEMMTACQGVGGPAHKARVLAEA |
| 64608 | gag | p24 | 1266-1434 | Q75002 | 2 | 2.1 | QDVKNWMTDTLLVQNANPDCKTILRALGPGASLEEMMTACQGVGGPAHKARVLAEA |
| 38473 | gag | p24 | 1272-1440 | P24736 | 2 | 2.1 | VKNWMTETLLVQNANPDCKSILRALGPGATLEEMMTACQGVGGPGHKARVLAEAMS |
| 19651 | gag | p24 | 1338-1506 | O91079 | 2 | 2.1 | KALGPGATLEEMMTACQGVGGPAHKARVLAEAMSQVQQPTTSVFAQRGNFKGIRKP |
| 77328 | gag | p7 | 1515-1683 | Q9QBZ2 | a | a.1, a.2 | NCGKEGHIARNCRAPRKRGCWKCGQEGHQMKDCTERQANFLGKMWPSNKGRPGNFL |
| 78373 | gag | p7 | 1515-1683 | Q9WC53 | a | a.1, a.2 | NCGKQGHIAKNCRAPRKKGCWKCGKEGHQMKDCTERQANFLGKIWPSSKGRPGNFL |
| 38476 | gag | p7 | 1530-1698 | P24736 | a | a.1, a.2 | GHLAKNCRAPRKKGCWKCGKEGHQMKDCTERQANFLGKIWPSNKGRPGNFPQSRPE |
| 23325 | gag | p7 | 1578-1746 | P04594 | a | a.1, a.2 | KCGKEGHQMKDCTERQANFLGKIWPSHKGRPGNFLQSRPEPTAPPAESFGFGEEIK |
| 19654 | gag | p7 | 1587-1755 | O91079 | a | a.1, a.2 | QEGHQMKDCKNEGRQANFLGKSWSPFKGRPGNFPQTTTRKEPTAPPLESYGFQEEK |
| 76945 | gag | p7 | 1587-1755 | Q9Q721 | a | a.,1 a.2 | QEGHQMKDCTGRQANFLGKIWPSSKGRPGNFPQKRLEPTAPPAESFGFGEEITPSP |
| 31703 | gag | p7 | 1590-1758 | P12495 | a | a.1, a.2 | EGHQLKDCTERQANFLGKIWPSHKGRPGNFLQSRPEPTAPPAESFGFGEEITPSQK |
| 20358 | gag | p7 | 1593-1761 | O93182 | a | a.1, a.2 | GHQMKDCTERQANFLGKIWPSSKGRPGNFLQSRPEPTAPPAESFGFGEEMTPSPKQ |
| 23308 | gag | p7 | 1593-1761 | P04592 | a | a.,1 a.2 | GHQLKDCTERQANFLGRIWPSHKGRPGNFLQSRPEPTAPPAESFGFGEEITPSQKQ |
| 27743 | gag | p7 | 1593-1761 | P0C1K7 | a | a.1, a.2 | GHQMKECTERQANFLGKIWPSNKGRPGNFLQNRTEPTAPPAESFGFGEEIAPSPKQ |
| 21869 | gag | p7 | 1596-1764 | P03347 | a | a.1, a.2 | HQMKDCTERQANFLGKIWPSYKGRPGNFLQSRPEPTAPPFLQSRPEPTAPPEESFR |
| 77329 | gag | p7 | 1599-1767 | Q9QBZ2 | a | a.2 | QMKDCTERQANFLGKMWPSNKGRPGNFLQNRPEPTAPPAESFGFGEEIAPSPKQEQ |
| 78374 | gag | p7 | 1599-1767 | Q9WC53 | a | a.2 | QMKDCTERQANFLGKIWPSSKGRPGNFLQSRPEPTAPPAESLGLGEEIPSPKQEPK |
| 35777 | gag | p7 | 1602-1770 | P18800 | a | a.2 | MKDCTERQANFLGKIWPSHKGRPGNFLQSRPEPTAPPAESFGFGEEITPSQKQEQK |
| 64595 | gag | p7 | 1605-1773 | Q75001 | a | a.2 | KDCTERQANFLGRLWPSNKGRPGNFLQSRPEPTAPPESLRPEPTAPPPESFRFEEA |
| 19353 | gag | p7 | 1611-1779 | O89291 | a | a.2 | CTERQANFLGKIWPSNKGRPGNFIQNRPEPSAPPAESFRFGEETTPSPKQEQKDEG |
| 77275 | gag | p7 | 1611-1779 | Q9QBY4 | a | a.2 | CTERQANFLGKIWPSHKGRPGNFLQSRPEPTAPPAESFGFGEEITPSPRQETKDKE |
| 22087 | pol | integrase | 4415-4583 | P03366 | 3 | 3.1 | QKQITKIQNFRVYYRDSRNPLWKGPAKLLWKGEGAVVIQDNSDIKVVPRRKAKIIR |
| 23273 | pol | integrase | 4436-4604 | P04588 | 3 | 3.1 | QNFRVYYRDNRDPIWKGPAKLLWKGEGAVVIQDNSDIKVVPRRKAKIIRDYGKQMA |
| 64646 | pol | integrase | 4439-4607 | Q75002 | 3 | 3.1 | NFRVYYRDSRDPIWKGPAKLLWKGEGAVVIQDNSDIKVVPRRKAKIIRDYGKQMAG |
| 35894 | env | gp120/41; V5, fusion peptide | 7133-7301 | P19549 | 6 | 6.1, 6.2 | TRDGGGDKNSTTEIFRPAGGNMKDNWRSELYKYKVVKIEPLGVAPTKAKRRVVQRE |
| 77391 | env | gp120/41; V5, fusion peptide | 7136-7304 | Q9QBZ8 | 6 | 6.1, 6.2 | RDGGNDNNTRTEETFRPGGGDMRDNWRSELYKYKVVQIEPLGIAPTRARRRVVQRE |
| 35748 | env | gp120/41; V5, fusion peptide | 7145-7313 | P18799 | 6 | 6.2 | GANNSSHETIRPGGGDMRDNWRSELYKYKVVKIEPIGVAPTKARRRVVEREKRAIG |
| 42484 | env | gp120/41; V5, fusion peptide | 7157-7325 | P35961 | 6 | 6.2 | TNGTEIFRPGGGDMRDNWRSELYKYKVVKIEPLGVAPTKAKRRVVQREKRAVGLGA |
| 77246 | env | gp120/41; V5, fusion peptide | 7163-7331 | Q9QBY2 | 6 | 6.2 | THNETFRPGGGDMRDNWRSELYKYKVVQIEPLGIAPTRARRRVVQREKRAVGLGAV |
| 18306 | env | gp120/41; V5, fusion peptide | 7166-7334 | O41803 | 6 | 6.2 | STEETFRPGGGDMRDNWRSELYKYKTVKIKSLGVAPTRARRRVVEREKRAVGLGAV |
| 76904 | env | gp120/41; V5, fusion peptide | 7190-7358 | Q9Q714 | 6 | 6.2 | DMRDNWRSELYKYKVVKIEPLGVAPTEARRRVVEREKRAVGMGAFFLGFLGAAGST |
| 77250 | env | gp41; HR2 | 7493-7661 | Q9QBY2 | 7 | 7.1 | QLRARILAVERYLKDQQLLGIWGCSGKLICTTNVPWNSSWSNKSWEEIWNNMTWME |
| 23125 | env | gp41; HR2 | 7508-7676 | P04579 | 7 | 7.1 | VLAVERYLRDQQLLGIWGCSGKLICTTTVPWNASWSNKSLNMIWNNMTWMQWEREI |
| 24026 | env | gp41; HR2 | 7514-7682 | P05882 | 7 | 7.1 | AVESYLKDQQLLGIWGCSGKHICTTTVPWNSSWSNKSLEEIWNNMTWIEWEREIDN |
| 22110 | env | gp41; HR2 | 7520-7688 | P03377 | 7 | 7.1 | ERYLKDQQLLGIWGCSGKLICTTAVPWNASWSNKSLEQIWNNMTWMEWDREINNYT |
| 23936 | env | gp41; HR2 | 7532-7700 | P05877 | 7 | 7.1 | KDQQLLGFWGCSGKLICTTTVPWNASWSNKSLDDIWNNMTWMQWEREIDNYTSLIY |
| 22140 | env | gp41; HR2 | 7538-7706 | P03378 | 7 | 7.1 | QQLLGIWGCSGKLICTTAVPWNASWSNKSLEDIWDNMTWMQWEREIDNYTNTIYTL |
| 17474 | env | gp41; HR2 | 7556-7724 | O12164 | 7 | 7.1 | WGCSGKLICTTAVPWNSSWSNRSQEDIWNNMTWMQWDREISNYTNTIYRLLEDSQN |

Legend for Supplemental Table 2. The table shows the features of the 43 peptides where higher antibody reactivity was associated with lower viral load. The following information is provided for each peptide: peptide identifier; HIV gene location; HIV protein location; HXB2 genomic coordinates (NCBI #NC_001802); UniProt identifier; cluster designation; epitope designation(s); amino acid sequence. Epitope sequences are shown in red font.

Abbreviations: gp: glycoprotein; HR: helical region; ID: identifier.

**Supplemental Figure 1. Peptide-level antibody responses and HIV viral load in the non-controller subset.**

Legend for Supplemental Figure 1: The plots show the association between the level of antibody reactivity to HIV peptides and HIV viral load as determined by linear regression. Data are shown for the subset of participants classified as non-controllers (n=64); this analysis included 1,183 HIV peptides that had significant antibody reactivity (adjusted fold change >1) for at least one participant in this subset. Panel A: The volcano plot shows the significance of the association between the level of antibody reactivity and viral load. The x-axis shows the estimated effect of antibody reactivity on viral load (estimated effect from the linear regression). Positive values indicate that higher levels of antibody reactivity were associated with higher viral loads; negative values indicate that higher levels of antibody reactivity were associated with lower viral loads. The y-axis shows the -log_10_ p-value for the association between the level of antibody reactivity and viral load. Each dot represents data for a single peptide. Panel B: The plot shows the same data visualized across the viral genome. The x-axis shows nucleotide position relative to genomic coordinates for the HIV HXB2 reference strain (NCBI #NC_001802). The y-axis shows the -log_10_ p-value for the association between antibody reactivity and viral load. Black dots indicate peptides for which higher antibody reactivity was associated with higher viral loads; red dots indicate peptides for which higher antibody reactivity was associated with lower viral loads. The genomic locations of the ten peptide clusters from Figure 2 are indicated by vertical gray lines.

Abbreviations: Kb: kilobase; VL: viral load.

**Supplemental Table 3. Epitope-level antibody responses and HIV viral load in the non-controller subset.**

| **Epitope** | **Estimated effect** | **p-value** |
| --- | --- | --- |
| 1.1 | -0.232 | 0.19 |
| 2.1 | -0.352 | **0.021** |
| a.1 | -0.189 | 0.23 |
| a.2 | -0.144 | 0.36 |
| 3.1 | -0.698 | **0.002** |
| 6.1 | -0.463 | **0.012** |
| 6.2 | -0.510 | **0.009** |
| 7.1 | -0.977 | **0.004** |

Legend for Supplemental Table 3. We evaluated whether an association between antibody reactivity and HIV viral load was observed at the epitope level for the subset of participants classified as non-controllers (n=64). The table shows the association between the level of antibody reactivity (log_10_ fold change) to the HIV epitopes described in Figure 4 and HIV viral load as determined by linear regression. Estimated effect and associated p-values were calculated using simple linear regression between antibody reactivity and viral load. The estimated effect indicates the change in viral load (log_10_ scale) associated with a unit increase in antibody reactivity (log_10_ scale). Negative values indicate that an increase in antibody reactivity was associated with a decrease in viral load. Statistically significant p-values are shown in bold font.

**Supplemental Figure 2: Aggregate antibody responses and HIV viral load in the non-controller subset.**


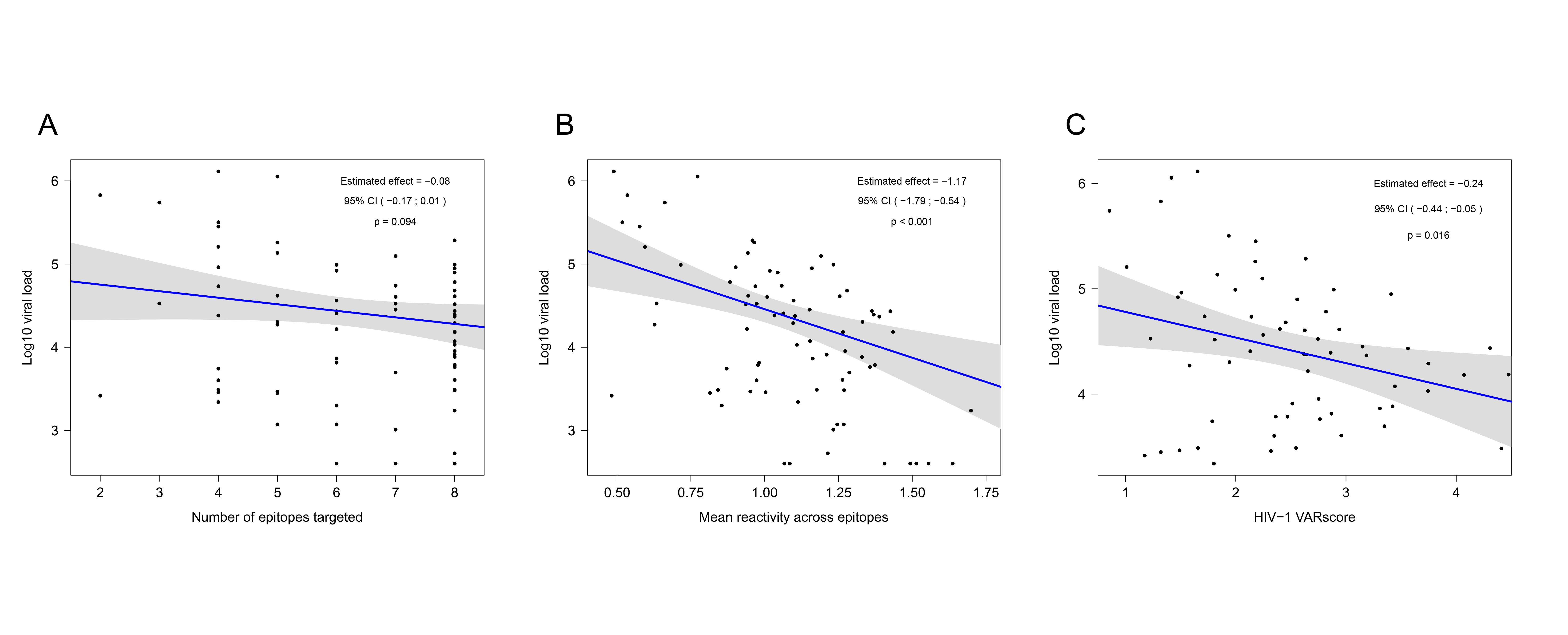


Legend for Supplemental Figure 2: The plots show the association between three aggregate measures of HIV antibody reactivity and HIV viral load, as determined by linear regression. Data are shown for the subset of participants classified as non-controllers (n=64). For each panel, each dot represents data for a single participant. The y-axes show the HIV viral load (log_10_ scale). The blue lines indicate the least squares regression lines. P-values indicate the significance of the associations as determined by linear regression. Grey regions show the 95% confidence bands for the mean antibody response. Panel A: Aggregate antibody reactivity was evaluated for the eight HIV epitopes shown in Figure 4. The x-axis shows the number of epitopes targeted (adjusted fold change >1). The estimated effect indicates the change in viral load (log_10_ scale) associated with one additional targeted epitope. Panel B: Mean antibody reactivity was evaluated across all eight HIV epitopes shown in Figure 4. The x-axis shows the mean antibody reactivity (log_10_ fold change) across all eight epitopes. The estimated effect indicates the change in viral load (log_10_ scale) associated with one unit increase in mean antibody reactivity (log_10_ scale). Panel C: The VARscore is a composite measure of the overall breadth and strength of antibody reactivity to all peptide targets across a viral genome, as measured by VirScan. The x-axis shows the HIV-1 VARscore. The estimated effect indicates the change in viral load (log_10_ scale) associated with one unit increase in HIV-1 VARscore.
